# Sympathetic Nervous Regulation of Calcium and Action Potential Alternans in the Intact Heart

**DOI:** 10.3389/fphys.2018.00016

**Published:** 2018-01-23

**Authors:** James Winter, Martin J. Bishop, Catherine D. E. Wilder, Christopher O'Shea, Davor Pavlovic, Michael J. Shattock

**Affiliations:** ^1^School of Cardiovascular Medicine and Sciences, King's College London, United Kingdom; ^2^Institute of Cardiovascular Sciences, College of Medicine and Dental Sciences, University of Birmingham, United Kingdom; ^3^Biomedical Engineering Department, King's College London, United Kingdom

**Keywords:** alternans, ventricular fibrillation, sympathetic nervous system, calcium transient, action potential duration, sarco(endo)plasmic reticulum ATPase, intact heart, optical mapping

## Abstract

**Rationale:** Arrhythmogenic cardiac alternans are thought to be an important determinant for the initiation of ventricular fibrillation. There is limited information on the effects of sympathetic nerve stimulation (SNS) on alternans in the intact heart and the conclusions of existing studies, focused on investigating electrical alternans, are conflicted. Meanwhile, several lines of evidence implicate instabilities in Ca handling, not electrical restitution, as the primary mechanism underpinning alternans. Despite this, there have been no studies on Ca alternans and SNS in the intact heart. The present study sought to address this, by application of voltage and Ca optical mapping for the simultaneous study of APD and Ca alternans in the intact guinea pig heart during direct SNS.

**Objective**: To determine the effects of SNS on APD and Ca alternans in the intact guinea pig heart and to examine the mechanism(s) by which the effects of SNS are mediated.

**Methods and Results**: Studies utilized simultaneous voltage and Ca optical mapping in isolated guinea pig hearts with intact innervation. Alternans were induced using a rapid dynamic pacing protocol. SNS was associated with rate-independent shortening of action potential duration (APD) and the suppression of APD and Ca alternans, as indicated by a shift in the alternans threshold to faster pacing rates. Qualitatively similar results were observed with exogenous noradrenaline perfusion. In contrast with previous reports, both SNS and noradrenaline acted to flatten the slope of the electrical restitution curve. Pharmacological block of the slow delayed rectifying potassium current (I_Ks_), sufficient to abolish I_Ks_-mediated APD-adaptation, partially reversed the effects of SNS on pacing-induced alternans. Treatment with cyclopiazonic acid, an inhibitor of the sarco(endo)plasmic reticulum ATPase, had opposite effects to that of SNS, acting to increase susceptibility to alternans, and suggesting that accelerated Ca reuptake into the sarcoplasmic reticulum is a major mechanism by which SNS suppresses alternans in the guinea pig heart.

**Conclusions**: SNS suppresses calcium and action potential alternans in the intact guinea pig heart by an action mediated through accelerated Ca handling and via increased I_Ks_.

## Introduction

Beat-to-beat oscillations in action potential duration (APD), termed APD alternans, are thought to be an important determinant of the induction of ventricular fibrillation (VF), by acting to promote functional conduction block and wave-break (Weiss et al., 2011). Mechanistically, APD alternans have been proposed to arise either as a function of the steepness of the electrical restitution or as secondary events driven by beat-to-beat oscillations in the amplitude of the intracellular Ca transient (Nolasco and Dahlen, 1968; Díaz et al., 2004).

It is known that APD and Ca alternans are affected by the sympathetic nervous system via β-adrenoceptor dependent signaling pathways. Sympathetic nerve stimulation (SNS) is reported to supress electrical (T-wave) and mechanical alternans associated with rapid atrial pacing in the canine heart (Euler et al., 1996). Similarly, β-adrenoceptor agonists are reported to suppress Ca transient alternans in isolated myocytes (Hüser et al., 2000; Florea and Blatter, 2012). However, studies in isolated rabbit hearts suggest that SNS promotes alternans by acting to steepen the slope of the electrical restitution curve (Ng et al., 2007).

Adrenergic stimulation exerts multiple effects on Ca handling and cardiac electrophysiology, including, an increase in the magnitude of the inward L-type Ca current, augmentation in the amplitude of the intracellular Ca transient, the acceleration of Ca uptake through the sarco/endoplasmic reticulum Ca^2+^-ATPase (SERCA) and increased conductance of the slow delayed rectifying K current (I_Ks_)—all of which could modulate Ca and APD alternans (Bers, 2002). Studies to date have largely focussed on the effects of adrenergic stimulation on Ca transient alternans in isolated myocytes treated with sympathomimetic agents, e.g., isoproterenol. Less is known about how activation of the regionally heterogeneous sympathetic innervation influences APD and Ca alternans in the intact heart with preserved cell-to-cell coupling, regional differences in ion channel expression and Ca handling properties. Currently, there are only two experimental reports examining the effects of SNS on alternans in the intact heart, which offer opposing conclusions (Euler et al., 1996; Ng et al., 2007). Reports in the rabbit, suggesting that SNS promotes alternans by steepening the slope of the electrical restitution curve, appear conflicted with more recent mechanistic insights from single cells, which place greater focus on instabilities of Ca handling. However, to date, there have been no studies of SNS on Ca alternans in the intact heart. The present study sought to address the hypothesis that SNS would act to suppress alternans. Experiments utilized voltage and Ca optical mapping for the simultaneous study of APD and Ca alternans in the intact guinea pig heart during electrical stimulation of the sympathetic nerves.

## Materials and methods

### Animal welfare

All procedures were undertaken in accordance with ethical guidelines set out by the UK Animals (Scientific Procedures) Act 1986 and Directive 2010/63/EU of the European Parliament on the protection of animals used for scientific purposes. Studies conformed to the Guide for the Care and Use of Laboratory Animals published by the U.S. National Institutes of Health under assurance number A5634-01. Studies were approved by the King's College London Animal Welfare and Ethical Review Board.

### Isolated innervated guinea pig heart and nerve stimulation

**Figure 1:**
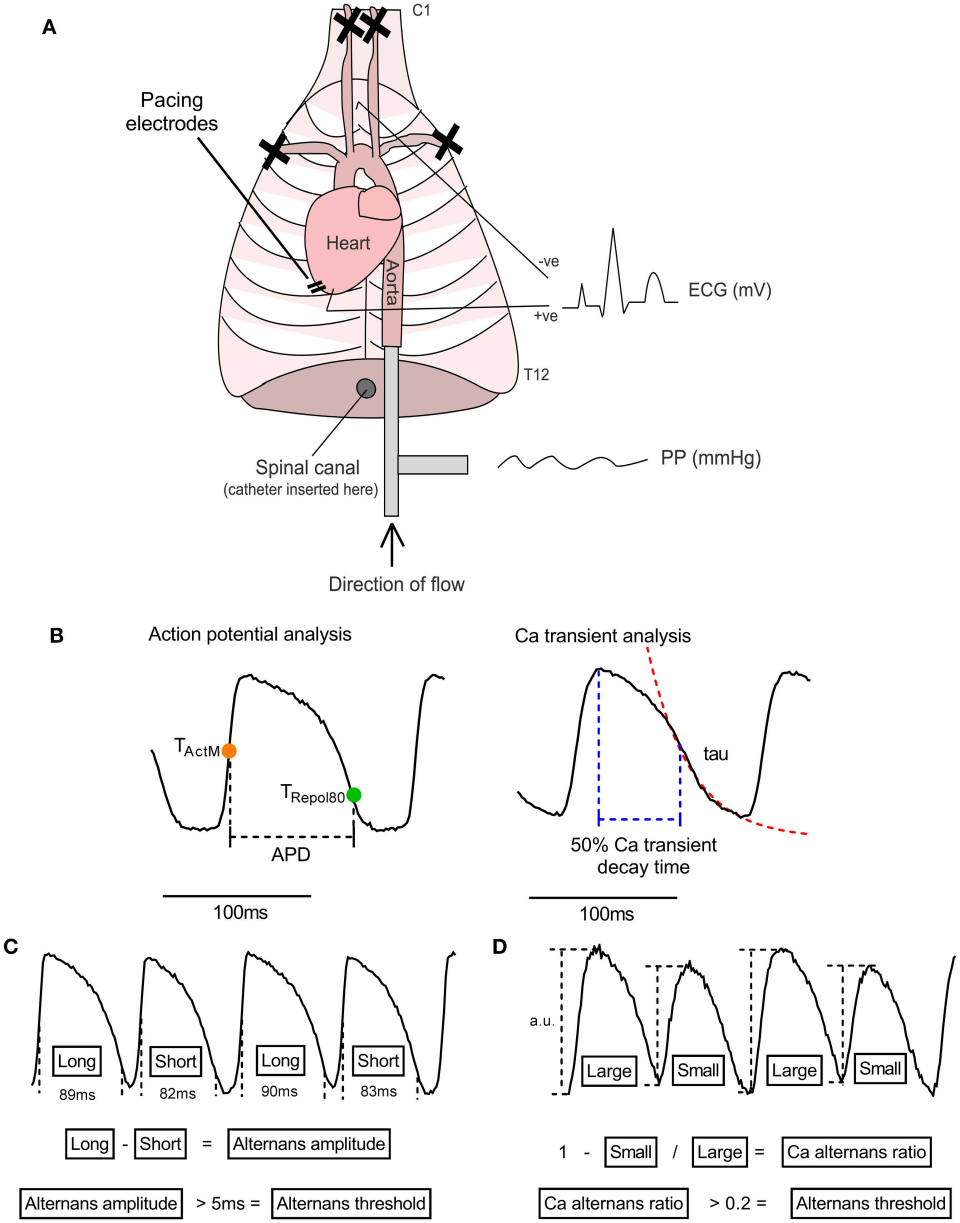
The isolated innervated guinea pig heart. **(A)** Illustrative representation of the isolated innervated guinea pig heart model. See text for details. PP, perfusion pressure; ECG, electrocardiogram. **(B)** Illustration of analysis parameters used for action potential and Ca transient recordings. Action potential duration (APD) was measured as the period between activation (midpoint of upstroke, T_ActM_) and 80% repolarisation (T_Repol80_). The rapid decay phase of the Ca transient was fitted to exponential function to derive the time constant (tau). Ca transient decay time was measured from peak of the Ca transient to 50% recovery. **(C,D)** Data analysis protocols for action potential and Ca transient amplitude alternans. For action potential duration, the maximum-minimum difference between pairs of beats is taken as the amplitude of alternans and the threshold for detection is set to 5 ms. For Ca transient alternans, the relative amplitude of the intracellular Ca transient, measured from the baseline preceding each beat, was used to calculate a Ca alternans ratio (see Equation 1). 0.1 is used as the threshold for Ca recordings.

Experiments were performed in adult male guinea pigs (400–650 g) utilizing an adaptation of the innervated rabbit heart preparation first described by Ng et al. (2001) (see Figure 1A). Animals were anesthetized (sodium pentobarbitone, 160 mg/kg i.p.), an incision was made below the diaphragm and the rib cage was resected on both sides. The descending aorta was isolated and cannulated, and the heart retrograde perfused with ice-cold oxygenated buffer (see below). The subclavian and carotid vessels were ligated, the heart and thorax isolated between cervical vertebrae 1 and thoracic vertebrae 12 and the preparation was transferred to a perfusion rig. Hearts were perfused in Langendorff mode through the descending aorta (perfusion pressure 65–75 mmHg, 37°C). Buffer solutions contained (in mmol/L): NaCl 114, KCl 4, CaCl 1.6, NaHCO_3_ 24, MgSO_4_ 1, NaH_2_PO_4_ 1.1, glucose 11.0, sodium pyruvate 1.0 and decamethonium bromide 0.01. Solutions were filtered using an in-line cellulose filter (5 μm pore diameter). An electrocardiogram (ECG) was recorded throughout the experiment (PowerLab 16/35 + Octal Bioamp, ADInstruments, Australia). Bi-lateral stimulation of efferent sympathetic nerves was conducted by insertion of a decapolar 5-french electrophysiological catheter into the spinal column, which was advanced to the level of the 5–7th cervical vertebrae. The distal poles of the catheter were attached to a constant voltage stimulator set at 40 V and with pulse duration of 2 ms (DS2A, Digitimer, UK). At the beginning of each experiment the response to nerve stimulation was tested and the frequency of electrical stimulation was varied (range 3–8 Hz) to give a steady state heart rate of between 320 and 340 bpm. Therein, parameters were kept constant during all experimental protocols and were stable for the duration of the experiment.

### Optical mapping

Hearts were uncoupled with blebbistatin (15 μmol/L) and stained with either di-8-anepps (1 mg/ml in DMSO; 200–300 μL), rh-237 (2.5 mg/ml in DMSO; 25–50 μL) or a combination of rh-237 (as previous) and rhod-2 AM (200 μg dissolved in 200 μL DMSO + 20 μL 20% pluronic F-127). Dyes were injected slowly into the perfusion line over a 5 to 10-min period. Excitation light was at 530 ± 25 nm, with emission light collected at >610 nm for di-8-anepps, >710 nm for rh-237 and 585 ± 20 nm for rhod-2. Hearts were imaged through an Olympus MVX10 stereomicroscope and signals recorded on Evolve Delta 512 × 512 pixel EMCCD cameras (500 Hz sampling rate, 64 × 64 pixels, 355 micron/pixel). Where appropriate, HMR 1556 (2 μmol/L in DMSO) and cyclopiazonic acid (CPA, 10 μmol/L in DMSO) were added directly to the perfusate and allowed to equilibrate for 10-min. Final DMSO concentration was <0.001% in all experiments. In some studies, noradrenaline (200 nmol/L) + ascorbic acid (50 μmol/L) was added directly to the perfusate.

### Pacing and VF induction

Hearts were paced from the left ventricular epicardial apex through silver bipolar pacing wires using a constant current stimulator (DS3, Digitimer, UK). Pulse duration and pulse amplitude were fixed at 2 ms and 5 mA, respectively. Initial studies utilized a dynamic pacing protocol in which hearts were paced at a cycle length for 100 beats and cycle length was then reduced in 10 ms steps every 10 beats until VF was induced or 2:1 block occurred. This pacing protocol was selected to limit extended periods of nerve stimulation, which lasted approximately 5-min per protocol. Where necessary, after VF induction, hearts were cardioverted by injection of high K+ solution (100 mmol/L) into the perfusion line. Hearts were allowed to recover for a minimum of 5-min between subsequent pacing protocols, or until heart rate and ECG parameters had stabilized. Due to substantive action potential prolongation, during HMR 1556 perfusion the dynamic pacing protocol was started from an initial pacing rate of 220 ms.

In some studies, the effects of noradrenaline on the Ca transient amplitude restitution relationship were measured using an extra-stimulus (S1–S2) pacing protocol, consisting of a 20-beat S1 train (cycle length = 200 ms) and a single S2 delivered at progressively shorter coupling intervals until loss of ventricular capture (10 ms step reduction from 200 ms). Ca transient restitution was measured as the % change in amplitude relative to the last S1 beat.

### Data analysis

Analysis was performed using custom MatLab scripts (MatLab, r2017b, MathWorks, USA). Data were processed with a Gaussian spatial filter (sigma = 1.5) and 5th order Savitzky-Golay temporal filter. Activation time was defined as the midpoint of the action potential upstroke. Repolarisation times were taken at 80% recovery to the resting membrane potential, measured from the peak to the end of the action potential. APD was taken as the difference between activation and repolarisation times (see Figure 1B).

APD alternans amplitude was calculated as the max-min for each pair of beats. A Ca alternans ratio was calculated from the difference in amplitude between large (and small Ca transients, according to Equation 1 (as per Wu and Clusin, 1997). An illustration of the analysis procedure for APD and Ca transient alternans can be found in Figures 1C,D.

(1) Ca alternans ratio=1−small/large

An alternans threshold of 5 ms and 0.1 was used for APD and Ca alternans, respectively.

APD and Ca transient restitution curves were fitted to Equation 2, where APD_ss_ is the APD at the slowest pacing rate, τ is the time constant, DI is the diastolic interval and b is the normalized minimum APD.

(2) $${\text{APD }=\text{ APD}}_{\text{ss}}^{*}[{1-\text{b}}^{*}\text{exp}(-\text{DI}/\tau )]$$

Presented data represent mean±SEM. Statistical comparisons were made by paired Student's *t*-test and two-way repeated measures ANOVA, with Tukey's *post-hoc* tests, as defined in the figure legends.

## Results

### The transition from concordant to discordant APD alternans underpins VF induction during dynamic pacing

**Figure 2:**
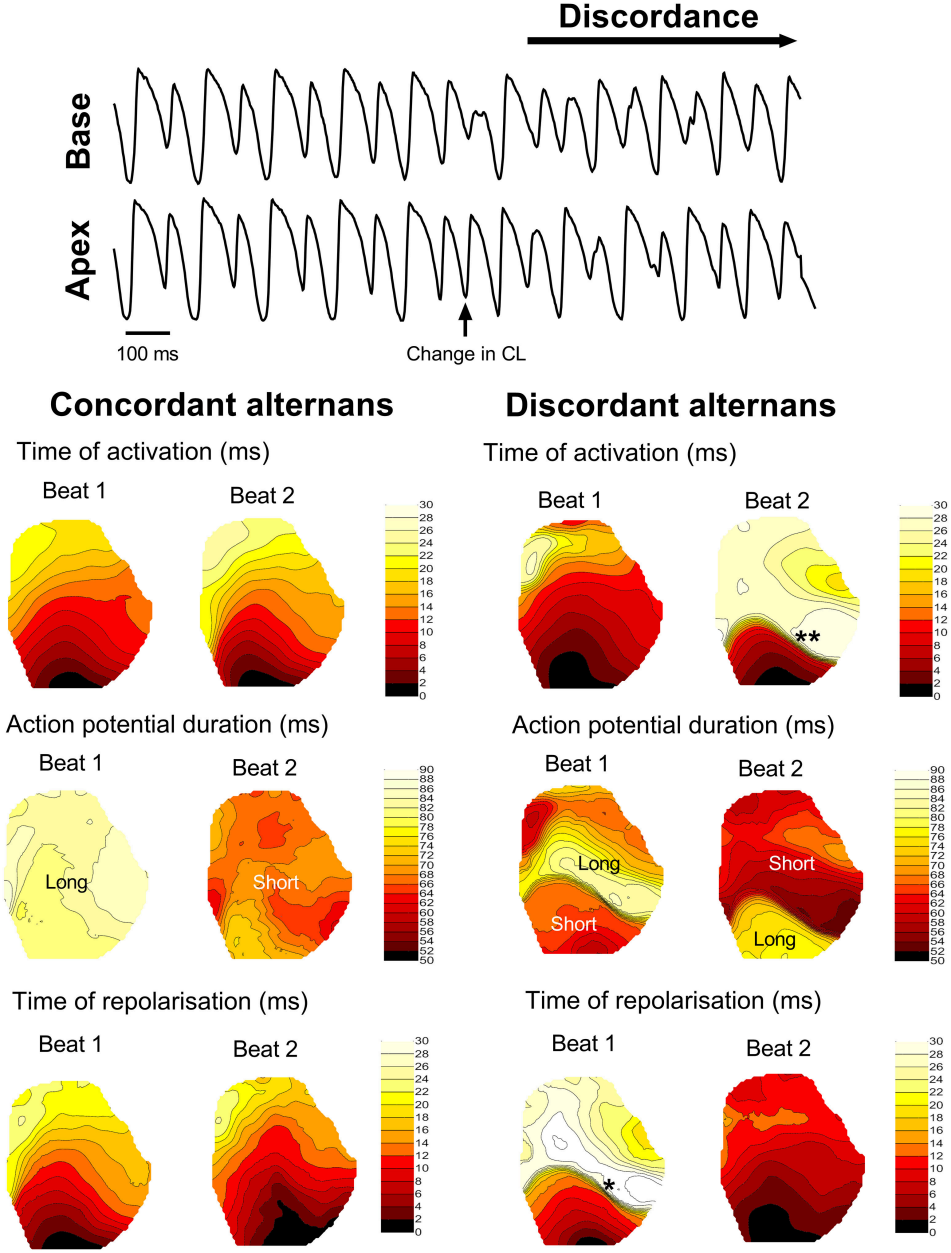
Transition from concordant to discordant alternans. Illustrative example of the transition from concordant to discordant alternans during dynamic pacing in the guinea pig heart. Representative traces from basal and apical regions are shown, along with representative isochronal maps of activation time, regional action potential duration and total repolarisation time across the guinea pig left ventricle during concordant and discordant alternans. Beats or regions with relatively long or short action potentials are labeled. ^*^denotes a region of tissue with a steep repolarisation gradient, secondary to discordant alternans, that results in regional condition slowing (^**^) on the next beat.

Dynamic (incremental) rapid-pacing of the guinea pig left ventricle was associated with oscillations in APD, which exhibited a pattern of long-short-long-short on a beat-to-beat basis. Alternans were initially spatially concordant (i.e., in-phase) across the ventricular surface, with a magnitude that increased as a function of pacing rate (left-hand side of Figure 2). With further increases in rate, spatially concordant alternans transitioned to become spatially discordant, where different regions of the ventricular myocardium exhibited counter-phase oscillations in APD (right-hand side of Figure 2), which was attributed to functional conduction slowing in regions with steep regional repolarisation gradients (see Figure 2). At a critical pacing rate, the resulting local differences in tissue repolarisation times (i.e., refractoriness), resulted in lines of unidirectional functional conduction block, retrograde activation, and the initiation of sustained re-entry/VF.

### SNS suppresses cardiac alternans in the guinea pig heart

**Figure 3:**
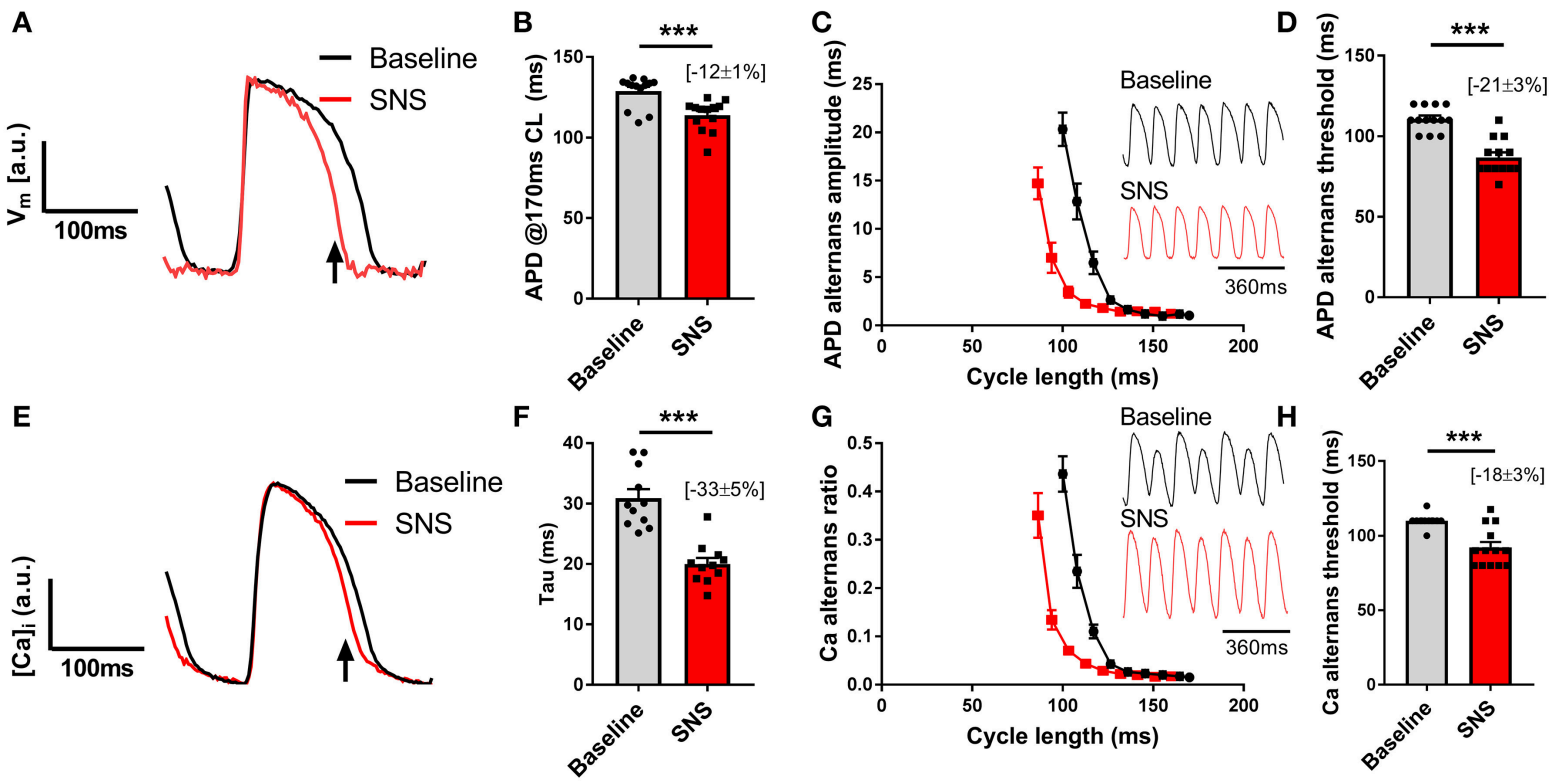
Effects of sympathetic nerve stimulation on action potential duration and Ca transient alternans. **(A,B)** Representative traces and data showing the change in action potential duration (APD) with sympathetic nerve stimulation (SNS). CL, cycle length. **(C)** Representative optical action potential recordings during pacing at a 120 ms cycle length and average cycle length relationships for the magnitude of APD alternans at baseline (black) and during SNS (red). **(D)** Data showing the change in the APD alternans threshold with SNS. **(E,F)** Representative trace showing the change in Ca transient kinetics with SNS and data demonstrating the effects of SNS on the time constant (tau) of Ca transient decay. **(G)** Representative optical Ca transient recordings during pacing at a 120 ms cycle length and average cycle length relationships for the magnitude of the Ca alternans ratio at baseline and during SNS. **(H)** Data showing the change in the Ca alternans threshold with SNS. Data taken from a 3 x 3 pixel region in the basal left ventricle. Data represent mean ± SEM. Paired Student's *t*-tests; ^***^p < 0.001. (*n* = 11–13 hearts).

**Figure 4:**
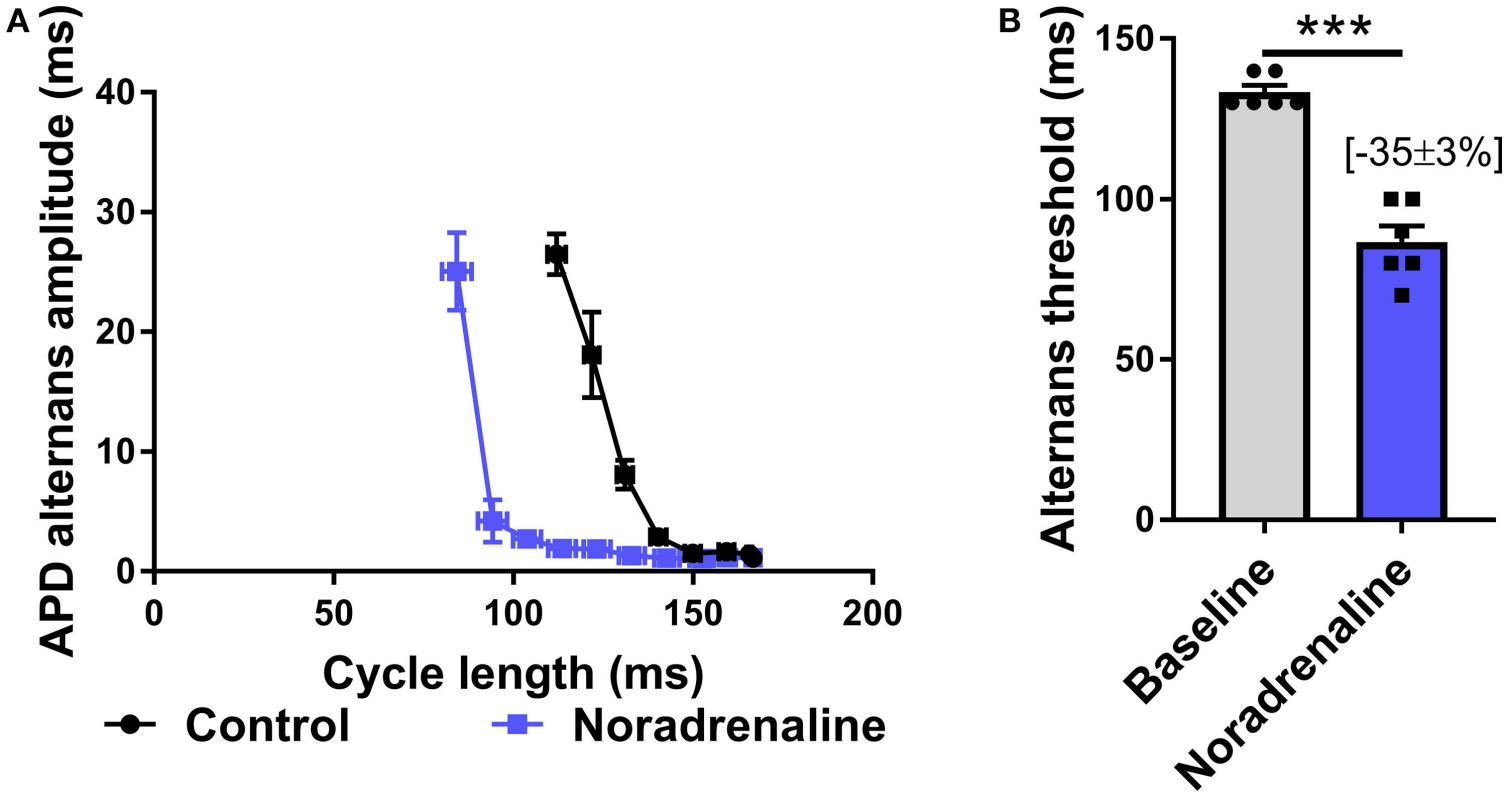
Effects of exogenous noradrenaline on action potential duration alternans. **(A)** Cycle length relationships of action potential duration (APD) alternans in control conditions (black) and with noradrenaline perfusion (blue). **(B)** Mean data of alternans threshold in both conditions. Data taken from a 3 × 3 pixel region in the basal left ventricle. Data represent mean ± SEM. Paired Student's *t*-test; ^***^p < 0.001. (*n* = 6 hearts).

Data presented in Figure 3 illustrate the effects of sustained SNS on dynamic pacing-induced APD and Ca transient alternans in the isolated innervated guinea pig heart. Stimulation of efferent sympathetic nerves resulted in rate-independent shortening of ventricular APD (Figures 3A,B) and the acceleration of Ca transient decay (i.e., decrease in tau; Figures 3E,F). Concomitant with these changes was the suppression of electrical and Ca alternans, as indicated by a leftward shift in the alternans cycle length relationships (Figures 3C,G) and a decrease in the alternans threshold (Figures 3D,H). Example recordings of rapid pacing induced APD and Ca transient alternans in the absence and presence of SNS are shown in Figures 3C,G, respectively. Addition of exogenous noradrenaline (200 nmol/L) to the perfusion buffer exerted qualitatively similar effects to those of SNS, acting to suppress APD alternans (Figure 4).

### SNS flattens the slope of the electrical restitution curve

**Figure 5:**
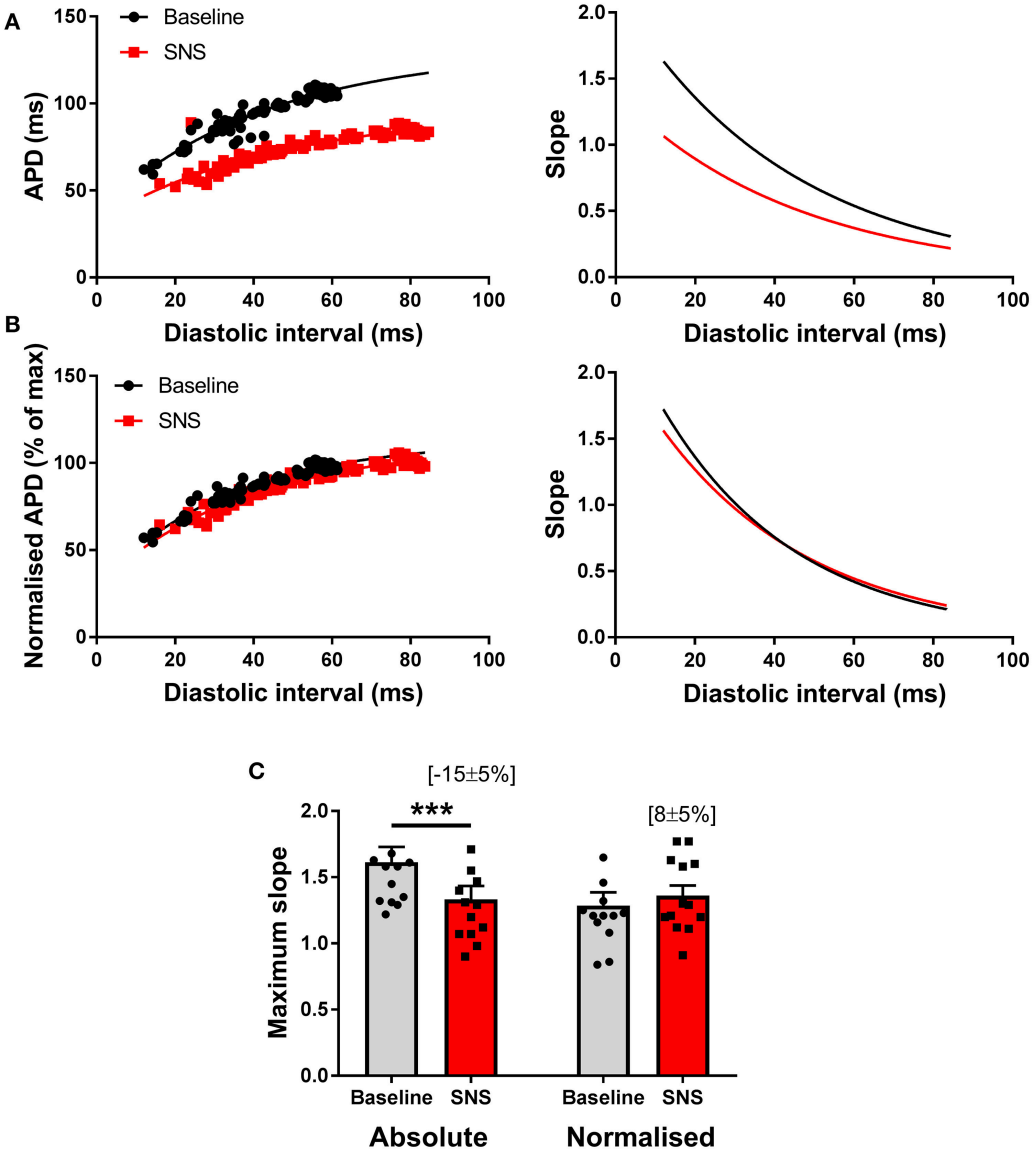
Modulation of the electrical restitution curve by sympathetic nerve stimulation. **(A)** Representative action potential duration (APD) restitution curves at baseline and during sympathetic nerve stimulation (SNS). The right panels show the first derivative of the exponential fit of each curve. **(B)** The same data with normalization to the maximum APD of each curve and the associated first derivative. **(C)** Data showing the effects of SNS on maximum slope of the APD restitution curve with and without normalization to account for differences in APD between conditions. Data represent mean ± SEM. Two-way ANOVA, with Tukey's *post-hoc* tests; ^***^p < 0.001. (*n* = 13 hearts).

Previous reports suggest that SNS acts to steepen the electrical restitution curve, and by this mechanism, augments pacing-induced APD alternans (Ng et al., 2007). However, this conflicts with our recent findings (Shattock et al., 2017), as we would predict that shortening of the ventricular action potential, would act to flatten, not steepen, the slope of the electrical restitution curve. To address this issue, we examined APD rate-adaptation with and without SNS. Data are presented in Figure 5. In accord with our previous observations, SNS and noradrenaline was associated with the flattening of the dynamic electrical restitution curve, secondary to shortening of the ventricular APD (Figures 5A,C). By normalizing the slope of the curves as a function of the APD at steady-state (i.e., the longest pacing cycle length) difference in slopes between conditions was abolished (Figures 5B,C).

### Mechanism(s) of action of SNS modulation of cardiac alternans

#### Role of APD adaptation

**Figure 6:**
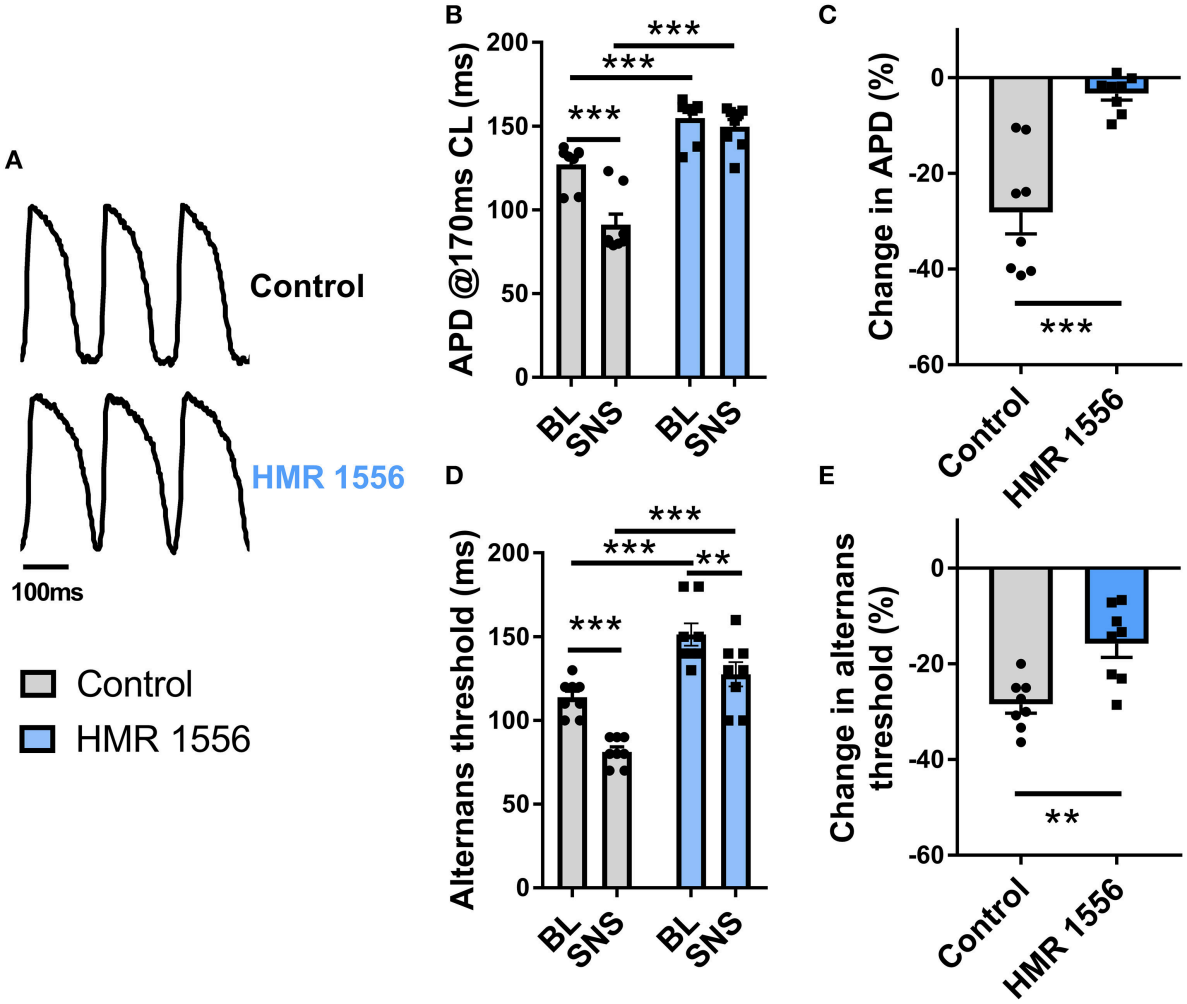
Effects of I_Ks_ block on alternans during sympathetic nerve stimulation. **(A)** Representative recordings of optical action potentials in control conditions and following perfusion of HMR 1556 (2 μmol/L). **(B)** Data showing ventricular action potential duration (APD) in control conditions and during sympathetic nerve stimulation (SNS) with and without HMR 1556. **(C)** Relative change in APD from baseline before and after perfusion of HMR 1556. **(D)** Data showing the change in the alternans threshold with SNS with and without HMR 1556. **(E)** Relative change in the alternans threshold before and after perfusion of HMR 1556. Data taken from a 3 × 3 pixel region in the basal left ventricle. Data represent mean ± SEM. Two-way ANOVA, with Tukey's *post-hoc* tests; ^**^p < 0.01, ^***^p < 0.001. Paired Student's *t*-test; ^**^p < 0.01, ^***^p < 0.001. (*n* = 8 hearts).

Augmentation of I_Ks_ is responsible for the rate-independent shortening of ventricular APD observed during adrenergic stimulation. By use of a selective inhibitor (HMR 1556, 2 μmol/L), we investigated the role of I_Ks_ in the effects of SNS. Data are presented in Figure 6. I_Ks_ block caused substantive prolongation of ventricular APD and prevented adaptation of the action potential during nerve stimulation (Figures 6A–C). HMR 1556 shifted the alternans cycle length relationship to the right, so that the threshold for alternans occurred at significantly slower pacing rates compared with control conditions (Figure 6D). In the presence of HMR 1556, SNS still suppressed alternans, however, the magnitude decrease in the alternans threshold was less compared with control measurements (~50% reduction in response to SNS; Figure 6E). This indicates that I_Ks_-mediated shortening of the ventricular action potential plays an important role in the suppression of cardiac alternans by SNS.

#### Role of Ca handling

**Figure 7:**
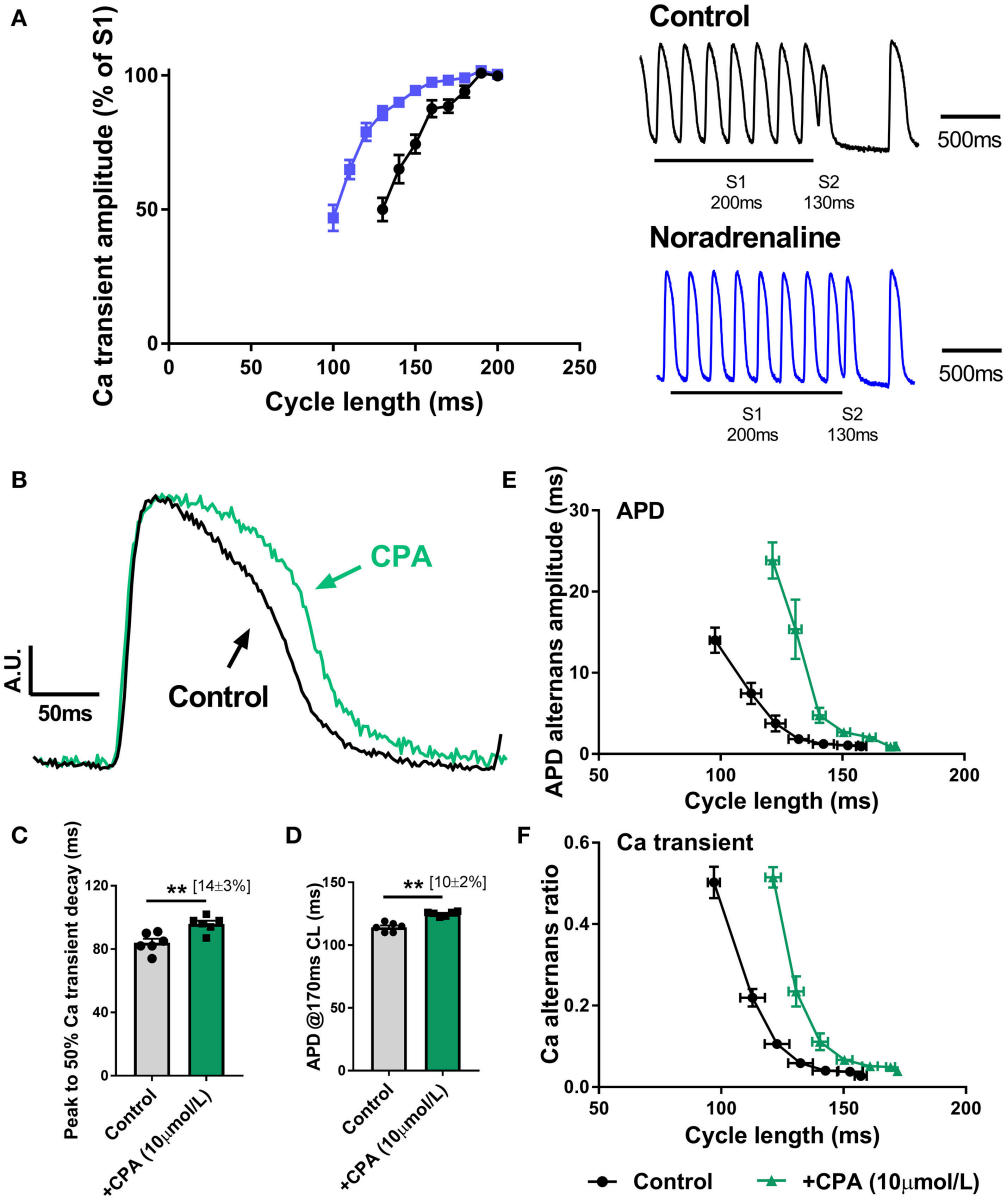
Role of altered Ca handling in the action of sympathetic nerve stimulation on cardiac alternans **(A)** S1S2 (extra-stimulus) Ca transient amplitude restitution relationships in the presence and absence of noradrenaline (200 nmol/L). Inset are representative traces of a single extra-stimulus (S2) at a 130 ms cycle length in the absence and presence of noradrenaline. **(B)** Amplitude-normalized intracellular Ca transients recorded in control conditions and with cyclopiazonic acid (CPA, 10 μmol/L). **(C,D)** Data on the effects of CPA on action potential duration (APD) and the time to 50% decay of the intracellular Ca transient. **(E,F)** Data on the cycle length relationships for APD and Ca amplitude alternans. Data taken from a 3 × 3 pixel region in the basal left ventricle. Paired Student's *t*-test; ^**^p < 0.01. Data are mean ± SEM. (*n* = 5–6 hearts).

As shown in Figure 3, SNS accelerates the rate of decay of the intracellular Ca transient, which is indicative of enhanced Ca reuptake via SERCA, a major target of adrenergic signaling pathways. In Figure 7A, it is shown that noradrenaline (equivalent to the action of SNS) causes a leftward shift in the Ca transient S1S2 (extra-stimulus) restitution relationship; an observation which is consistent with the notion that Ca alternans are a function of the rate of Ca extrusion from the cytosol (see Discussion). During SNS, Ca uptake into the SR is accelerated, and so the amplitude of the Ca transient is preserved over a broader range of pacing rates (Figure 7A). Alternans arise when the rate of SR Ca recovery cannot keep up with the underlying beating rate. Thus, the threshold for alternans occurs at faster rates (i.e., shorter cycle lengths) during adrenergic stimulation (Figures 3G,H). In support of this argument, we found that perfusion of a submaximal concentration of the CPA (10 μM), an inhibitor of SERCA, exerted opposite effects to those observed with SNS. Perfusion of CPA was associated with slowing of the decay of the intracellular Ca transient (Figures 7B,C), prolongation of the ventricular action potential (Figure 7D) and a rightward shift in the cycle length relationship for both APD and Ca alternans (Figures 7E,F). Notably, the suppression of alternans by SNS was preserved in the presence of CPA (data not shown), but this was predicted, as adrenergic stimulation affects the K_m_ of SERCA, whereas CPA inhibits the pump (i.e., reduces pump density). Thus, SNS would still be expected to accelerate Ca uptake in the presence of submaximal concentrations of CPA.

In summary, both enhanced rates of cytosolic Ca extrusion and shortening of the ventricular action potential, via increased I_Ks_, contribute to the suppression of cardiac alternans by SNS.

## Discussion

The results of the present study indicate that SNS suppresses APD and Ca alternans in the intact guinea pig heart. SNS acts to shift the threshold for alternans to a lower set point, so that faster beating rates are required to induce instabilities of Ca cycling and membrane potential. Our observations are confirmatory of previous studies in isolated myocytes treated with sympathomimetic agents (Hüser et al., 2000; Florea and Blatter, 2012), as well as indirect observations in the dog (i.e., mechanical and T-wave alternans) (Euler et al., 1996), but differ from the reported effects of SNS in the rabbit heart (i.e., augmented APD alternans secondary to steepening of the electrical restitution curve). The results of the present study suggest that in the guinea pig heart, SNS suppresses alternans by two main mechanisms of action; (1) accelerated reuptake of cytosolic Ca into the SR and (2) I_Ks_-mediated shortening of the ventricular action potential. In this paradigm, Ca-dependent mechanisms are the major driver of alternans, whilst the slope of the restitution curve plays a smaller modulatory role.

### Cellular mechanisms of alternans and modulation by adrenergic stimulation

Cardiac alternans have received substantial experimental and clinical interest due to the strong association between alternans, cardiac pathology, and sudden death. Understanding of the mechanistic basis for alternans has evolved from an initial focus on voltage-dependent mechanisms (i.e., the slope of the electrical restitution curve), toward the more recent recognition of Ca cycling instabilities as the primary driving mechanism (Weiss et al., 2011). In what is now a classical study, Diaz et al. first proposed that the steep Ca load-release relationship of the sarcoplasmic reticulum was the primary basis of Ca (and, indirectly, APD) alternans (Díaz et al., 2004). However, this idea was subsequently challenged by observations that Ca transient alternans can occur in the absence of oscillations in the diastolic Ca level of the SR (Picht et al., 2006). It has since been proposed that Ca transient alternans are a function of the recovery from inactivation of the RyRs, which have an intrinsic refractory period that is modulated by cytosolic and luminal Ca levels (Picht et al., 2006; Shkryl et al., 2012; Wang et al., 2014; Qu et al., 2016). Recent theoretical modeling based on the emergent properties of the cardiac couplon network, indicates that the key factors responsible for alternans are the random nature of Ca sparks (RyR openings), RyR refractoriness and recruitment of neighboring Ca-release units (the 3R theory) (Weiss et al., 2011; Qu et al., 2016). In this paradigm, the steep load-release relationship arises intrinsically from the myocyte couplon network, as recently proposed by Qu et al. (2016). Thus, Ca transient alternans involve several interacting and interdependent mechanisms. For example, in studies of SR Ca cycling in isolated rabbit hearts, Wang et al. reported that alternans of SR Ca-release precede changes in diastolic SR Ca (Wang et al., 2014). However, at faster pacing rates, diastolic SR Ca oscillates on a beat-by-beat basis. This suggests that RyR refractoriness is the principal driver of Ca transient alternans, but at faster rates, the steep load-release relationship of the SR is engaged. Thus, the study of Wang et al. appears to be in keeping with the unified model of Qu et al. (2016).

In the present study, we report two major mechanisms of action for the sympathetic nervous modulation of cardiac alternans; (1) enhanced cytosolic Ca extrusion and (2) I_Ks−_mediated shortening of the ventricular action potential. In the basic framework discussed above, the first mechanism is explained by accelerated Ca uptake into the SR, resulting in a more rapid rate of recovery of RyR refractoriness (which is sensitive to both cytosolic and luminal Ca levels). At any given cycle length, this means that a greater proportion of RyRs are fully recovered in time for the next beat, which prevents oscillations in Ca release. Concomitantly, the Ca load of the SR will also recover more quickly, preventing oscillations of Ca load and release (as in Diaz et al.). Both mechanisms (or a combination) can adequately explain why SNS, and noradrenaline, act to suppress alternans, and is supported by our experimental observations, namely, the leftward shift in the APD and Ca alternans cycle length relationships and leftward shift in the Ca transient S1S2 (extra-stimulus) restitution curve. Indeed, there is already good evidence that SERCA is a critical regulator of alternans. For example, Cuttler et al. showed that targeted overexpression of genes for SERCA2a in isolated guinea pig myocytes and intact hearts acts to suppresses both Ca and APD alternans (Cutler et al., 2009). Similarly, Laurita et al. described regional heterogeneities in SERCA expression that correlate with the magnitude of alternans across the ventricular wall of the canine heart (Laurita et al., 2003). In the present study, pharmacological inhibition of SERCA exerted the opposite effect to that of SNS and noradrenaline, which indirectly supports the importance of altered SERCA activity in suppression of APD and Ca alternans during adrenergic stimulation.

The second mechanism by which adrenergic-stimulation suppresses APD alternans is through I_Ks_-mediated shortening of the ventricular action potential. Two potential mechanisms could explain this observation. Firstly, the slope of the restitution curve is largely determined by the APD at steady-state (Shattock et al., 2017) and shortening of the action potential with SNS acts to flatten this relationship. Altered restitution kinetics, secondary to I_Ks_-mediated action potential adaptation, could perceivably dampen the magnitude of APD alternans. Notably, this does not require that the slope of the curve be >1, as in the classical proposal of Nolasco and Dahlen (Nolasco and Dahlen, 1968). Rather, a shorter action potential is predicted to exhibit smaller alternans than an action potential of longer initial duration, simply because it is shorter in the first place. This is an inherent physical property of the action potential that arises from the non-linear dependence of APD on repolarisation rate (see Zaza, 2010; Winter and Shattock, 2016). The potential second mechanism is that I_Ks_-mediated shortening of the action potential may indirectly regulate intracellular Ca handling. It is known that the duration of the action potential influences cellular Ca influx and efflux, and prolonging the action potential results in an increase in Ca load and slowing in the rate of decay of the Ca transient (Bouchard et al., 1995). I_Ks_-mediated shortening of the action potential may therefore enhance cytosolic Ca extrusion by limiting Ca entry via L-type Ca current and/or by modulating Ca flux through the sarcolemmal sodium-Ca exchanger.

The results of the present study differ from previous reports in the rabbit heart, which suggest that SNS leads to a steepening of the electrical restitution curve, an increase in the magnitude of APD alternans and a decrease in the ventricular fibrillation threshold (a pseudo-quantitative measure of susceptibility to ventricular fibrillation). However, we found that SNS acts to supress APD alternans, in line with earlier reports on mechanical and T-wave alternans in dogs (Euler et al., 1996) and supported by several reports in isolated myocytes treated with sympathomimetic agents (Hüser et al., 2000; Florea and Blatter, 2012). Moreover, our findings are in keeping with more recent thinking on the primacy of Ca cycling instabilities as a driver for APD alternans. However, we cannot discount that the difference between our study and the study of Ng et al. reflect the species used (guinea pig vs. rabbit) or methodological differences between studies (optical mapping vs. monophasic action potential recordings). Notably, there are measurable differences in electrophysiology between experimental mammalian species, most markedly between smaller and larger species (rat/mouse vs. rabbit/dog), but the rabbit is also known to exhibit a distinct biphasic restitution relationship (Szigligeti et al., 1998). It is feasible that species-specific differences could account for the discord between the effects of SNS on alternans in rabbits and guinea pigs (and other species). Whether the results of our experiments in the guinea pig are generalizable to other species is not known and requires further study.

### Study limitations and technical discussion

This study has several limitations. Firstly, because we used Rhod-2, a single wavelength fluorescent indicator, the presented values for Ca transient alternans are only relative and cannot be reliably calibrated in the isolated heart. However, this is unlikely to impact on the main conclusions of the study. Furthermore, the use of uncoupling agents in optical mapping is a recognized technical limitation, with the potential to adversely affect cardiac electrophysiology (Brack et al., 2013). In the present study, we observed no obvious electrophysiological changes following blebbistatin perfusion in the guinea pig heart, in keeping with reports in other species (Fedorov et al., 2007).

In the present study, alternans were only observed at pacing cycle lengths shorter than 120 ms, which differs from the classical studies of Pastore et al., where alternans were observed in guinea pig hearts paced at slower rates (Pastore et al., 1999). This disparity most likely reflects that Pastore et al. performed their studies at relatively cool temperatures (27°C vs. 37°C) and with lower buffer Ca then used presently (1.2 mmol/L vs. 1.6 mmol/L), both of which would prolong APD and so shift the alternans threshold to slower rates. In addition, to avoid sustained periods of nerve stimulation, which could cause rundown of nerve responses, we utilized a dynamic pacing protocol, whereas previous studies have used more sustained pacing periods (~1 min per cycle length).

## Conclusion(s)

SNS suppresses APD and Ca alternans in the intact guinea pig heart by accelerated intracellular Ca handling and via I_Ks_-mediated shortening of the ventricular action potential.

## Author contributions

JW was responsible for the central hypothesis, design and implementation of the study, collection and analysis of most experimental data and primarily responsible for drafting of the manuscript. CW, MB, and MS contributed to the study design, analysis of experimental data and drafting of the manuscript. CW also assisted with the collection and analysis of the experimental data. CO and DP provided expertise in the analysis of the optical mapping datasets, the interpretation of the results and drafting of the final manuscript.

### Conflict of interest statement

The authors declare that the research was conducted in the absence of any commercial or financial relationships that could be construed as a potential conflict of interest.

## Abbreviations

APD: action potential duration
SERCA: sarco(endo)plasmic reticulum ATPase
SR: sarcoplasmic reticulum
SNS: sympathetic nerve stimulation.
